# Out of DSM: Depathologizing Homosexuality

**DOI:** 10.3390/bs5040565

**Published:** 2015-12-04

**Authors:** Jack Drescher

**Affiliations:** 1 440 West 24 Street, #1A, New York, NY 10011, USA; E-Mail: jackdreschermd@gmail.com; Tel.: +1-212-645-2232; 2 Department of Psychiatry, New York Medical College, 20 Hospital Rd, Valhalla, New York, NY 10595, USA; 3 Postdoctoral Program in Psychotherapy and Psychoanalysis, New York University, 547 La Guardia Place, New York, NY 10012, USA; 4 William A. White Institute, 20 West 74th Street, New York, NY 10023, USA

**Keywords:** American Psychiatric Association (APA), diagnosis, Diagnostic and Statistical Manual (DSM), gender beliefs, gender binaries, homosexuality, psychiatry

## Abstract

In 1973, the American Psychiatric Association (APA) removed the diagnosis of “homosexuality” from the second edition of its Diagnostic and Statistical Manual (DSM). This resulted after comparing competing theories, those that pathologized homosexuality and those that viewed it as normal. In an effort to explain how that decision came about, this paper reviews some historical scientific theories and arguments that first led to the placement of homosexuality in DSM-I and DSM-II as well as alternative theories that eventually led to its removal from DSM III and subsequent editions of the manual. The paper concludes with a discussion of the sociocultural aftermath of that 1973 decision.

## 1. Introduction

In 1973, the American Psychiatric Association (APA) removed the diagnosis of “homosexuality” from the second edition of its Diagnostic and Statistical Manual (DSM) [1,2]. This resulted after comparing competing theories, those that pathologized homosexuality and those that viewed it as normal [3,4,5,6]. In an effort to explain how that decision came about, this paper reviews some historical scientific theories and arguments that first led to the placement of homosexuality in DSM-I [7] and DSM-II [8], as well as alternative theories, that eventually led to its removal from DSM III [9] and subsequent editions of the manual [10,11,12,13]. The paper concludes with a discussion of the sociocultural aftermath of that 1973 decision. 

## 2. Theories of Homosexuality

It is possible to formulate a descriptive typology of etiological theories of homosexuality throughout modern history in which they generally fall into three broad categories: *pathology, immaturity, and normal variation* [14,15,16].

### 2.1. Theories of Pathology

These theories regard adult homosexuality as a disease, a condition deviating from “normal,” heterosexual development [17]. The presence of atypical gender behavior or feelings are symptoms of the disease or disorder to which mental health professionals need to attend. These theories hold that some internal defect or external pathogenic agent causes homosexuality and that such events can occur pre- or postnatally (*i.e.*, intrauterine hormonal exposure, excessive mothering, inadequate or hostile fathering, sexual abuse, *etc*.). Theories of pathology tend to view homosexuality as a sign of a defect, or even as morally bad, with some of these theorists being quite open about their belief that homosexuality is a social evil. For example, psychiatrist and psychoanalyst Edmund Bergler infamously wrote in a book for general audiences, “I have no bias against homosexuals; for me they are sick people requiring medical help... Still, though I have no bias, I would say: Homosexuals are essentially disagreeable people, regardless of their pleasant or unpleasant outward manner... [their] shell is a mixture of superciliousness, fake aggression, and whimpering. Like all psychic masochists, they are subservient when confronted with a stronger person, merciless when in power, unscrupulous about trampling on a weaker person” [18], (pp. 28–29). 

### 2.2. Theories of Immaturity

These theories, usually psychoanalytic in nature, regard expressions of homosexual feelings or behavior at a young age as a normal step toward the development of adult heterosexuality [19,20]. Ideally, homosexuality should just be a passing phase that one outgrows. However, as a “developmental arrest,” adult homosexuality is equated with stunted growth. Those who hold these theories tend to regard immaturity as relatively benign, or at least not as “bad” compared to those who theorize that homosexuality is a form of psychopathology.

### 2.3. Theories of Normal Variation 

These theories treat homosexuality as a phenomenon that occurs naturally [21,22,23,24]. Such theories typically regard homosexual individuals as born different, but it is a natural difference affecting a minority of people, like left-handedness. The contemporary cultural belief that people are “born gay” is a normal variation theory. As these theories equate the normal with the natural, they define homosexuality as good (or, at baseline, neutral). Such theories see no place for homosexuality in a psychiatric diagnostic manual.

## 3. Gender Beliefs

It is rare to find a theory of homosexuality that does not draw upon *gender beliefs* that contain implicit cultural ideas about the “essential” qualities of men and women [14,16,25]. “Real men” and “real women” are powerful cultural myths with which everyone must contend. People express gender beliefs, their own and those of the culture in which they live, in everyday language as they either indirectly or explicitly accept and assign gendered meanings to what they and others do, think, and feel. Gender beliefs touch upon almost every aspect of daily life, including such mundane concerns as what shoes men should wear or “deeper” questions of masculinity such as whether men should openly cry or sleep with other men. Gender beliefs are embedded in questions about what career a woman should pursue and, at another level of discourse, what it would mean if a professional woman were to forego rearing children or pursue a career more aggressively than a man. 

Gender beliefs are usually based upon *gender binaries.* The most ancient and well known is the male/female binary. However there is also the 19th century binary of homosexuality/heterosexuality (or gay/straight in the 20th century) and the emerging 21st century binary of transgender/cisgender. It should be noted that binaries are not confined to popular usage. Many scientific studies of homosexuality contain implicit (and often explicit) binary gender beliefs as well. For example, the *intersex hypothesis of homosexuality* [26,27] maintains that the brains of homosexual individuals exhibit characteristics that would be considered more typical of the other sex. The essentialist gender belief implicit in the intersex hypotheses is that an attraction to women is a masculine trait, which in the case of Sigmund Freud [28], for example (also see below), led to his theory that lesbians have a masculine psychology. Similarly, biological researchers have presumed gay men have brains that more closely resemble those of women [29] or are recipients of extra fragments of their mothers’ X (female) chromosomes [30].

Gender beliefs usually only allow for the existence of two sexes. To maintain this gender binary, most cultures traditionally insisted that every individual be assigned to the category of either man or woman at birth and that individuals conform to the category to which they have been assigned thereafter. The categories of “man” and “woman” are considered to be mutually exclusive, although there are exceptions, as in Plato’s *Symposium* and some Native American cultures [31]. (Also see Fausto-Sterling [32,33,34] for a scientist’s thoughtful criticisms of gender binaries). These beliefs underlie mid-20th century theories that children born with anomalous genitalia had to immediately undergo unnecessary medical surgeries in order to reduce their parents’ anxieties about whether they were boys or girls [25,34,35].

Rigid gender beliefs usually flourish in fundamentalist, religious communities where any information or alternative explanations that might challenge implicit and explicit assumptions are unwelcome. When entering the realms of gender and sexuality, it is not unusual to encounter another form of binary thinking: “morality tales” about whether certain kinds of thoughts, feelings, or behaviors are “good or bad” or, in some cases, whether they are “good or evil” [14,15,16]. The good/bad binary is not confined to religion alone, as the language of morality is inevitably found, for example, in theories about the “causes” of homosexuality. For in the absence of certitude about homosexuality’s “etiology,” binary gender beliefs and their associated moral underpinnings frequently play a role in theories about the causes and/or meanings of homosexuality. When one recognizes the narrative forms of these theories, some of the moral judgments and beliefs embedded in each of them become clearer.

## 4. Early Theorists of Homosexuality

For much of Western history, official pronouncements on the meanings of same-sex behaviors were primarily the province of religions, many of which deemed homosexuality to be morally “bad” [36]. However, as 19th century Western culture shifted power from religious to secular authority, same-sex behaviors, like other “sins,” received increased scrutiny from the law, medicine, psychiatry, sexology, and human rights activism. Eventually, religious categories like *demonic possession, drunkenness*, and *sodomy* were transformed into the scientific categories of *insanity, alcoholism*, and *homosexuality.*

Thus, the modern history of homosexuality usually begins in the mid-19th century, most notably with the writings of Karl Heinrich Ulrichs [21]. Trained in law, theology, and history, he might be considered an early gay rights advocate who wrote a series of political tracts criticizing German laws criminalizing same sex relationships between men. He hypothesized that some men were born with a woman’s spirit trapped in their bodies and that these men constituted a *third sex* he named *urnings.* He also defined a woman who we would today call a lesbian as *urningin*, a man’s spirit trapped in the body of a woman. 

In 1869, Hungarian journalist Károli Mária Kertbeny first coined the terms “homosexual” and “homosexuality” in a political treatise against Paragraph 143, a Prussian law later codified in Germany’s Paragraph 175 that criminalized male homosexual behavior [37]. Kertbeny put forward his theory that homosexuality was inborn and unchangeable, arguments that it was a normal variation, as a counterweight against the condemnatory moralizing attitudes that led to the passage of sodomy laws. 

Richard von Krafft-Ebing, a German psychiatrist, offered an early theory of pathology, describing homosexuality as a “degenerative” disorder. Adopting Kertbeny’s terminology, but not his normalizing beliefs, Krafft-Ebing’s 1886 *Psychopathia Sexualis* [17] viewed unconventional sexual behaviors through a lens of 19th century Darwinian theory: non-procreative sexual behaviors, masturbation included, were regarded as forms of psychopathology. In an ironic twist of the modern “born gay” theory, Krafft-Ebing believed that although one might be born with a homosexual predisposition, such inclinations should be considered a congenital disease. Krafft-Ebing was influential in disseminating among the medical and scientific communities both the term “homosexual” as well as its author’s view of homosexuality as a psychiatric disorder. *Psychopathia Sexualis* would presage many of the pathologizing assumptions regarding human sexuality in psychiatric diagnostic manuals of the mid-20th century.

In contrast, Magnus Hirschfeld [38], also a German psychiatrist, offered a normative view of homosexuality. Hirschfeld, an openly homosexual physician and sex researcher, was a leader of the German homophile movement of his time as well as the standard bearer of Ulrich’s [21] 19th century *third sex* theories. 

## 5. Psychoanalytic Theorizing

Directly refuting Hirschfeld’s theories of normal variation and Krafft-Ebing’s theory of pathology, Sigmund Freud [19] put forward an alternative theory that would also find its way into the popular imagination. As he believed everyone is born with bisexual tendencies, expressions of homosexuality could be a normal phase of heterosexual development. This belief in innate bisexuality did not allow for the possible existence of Hirschfeld’s third sex: “Psychoanalytic research is most decidedly opposed to any attempt at separating off homosexuals from the rest of mankind as a group of special character” [19], (p. 145n). Further, Freud argued that homosexuality could not be a “degenerative condition” as Krafft-Ebing maintained because, among other reasons, it was “found in people whose efficiency is unimpaired, and who are indeed distinguished by specially high intellectual development and ethical culture” [19], (p. 139). Instead, Freud saw expressions of adult homosexual behavior as caused by “arrested” psychosexual development, a theory of immaturity. Toward the end of his life, Freud wrote “Homosexuality is assuredly no advantage, but it is nothing to be ashamed of, no vice, no degradation; it cannot be classified as an illness; we consider it to be a variation of the sexual function, produced by a certain arrest of sexual development” [39], (p. 423). This belief made him pessimistic about efforts to change a homosexual orientation to a heterosexual one: “In general, to undertake to convert a fully developed homosexual into a heterosexual does not offer much more prospect of success than the reverse, except that for good practical reasons the latter is never attempted” [28], (p. 151).

Yet after Freud’s death in 1939, most psychoanalysts of the next generation came to view homosexuality as pathological. They offered a revised understanding of homosexuality as well as psychoanalytic “cures” that had eluded the field’s founder. Their views were based on the theories of Sandor Rado [40,41], a Hungarian émigré to the United States whose theories had a significant impact on American psychiatric and psychoanalytic thought in the mid-20th century. Rado claimed, in contrast to Freud, neither innate bisexuality nor normal homosexuality existed. Heterosexuality was the only biological norm and homosexuality reconceptualized as a “phobic” avoidance of the other sex caused by inadequate parenting. Rado’s theorizing informed the work of Bieber *et al*. [42] and Socarides [43], analysts whose claims of psychoanalytic “cures” of homosexuality were broadly accepted by their professional community although never verified in any meaningful or empirical way (cf. Moor [44]; Tripp [45]). 

In the mid-20th century American psychiatry was greatly influenced at the time by these psychoanalytic perspectives. Consequently, in 1952, when APA published the first edition of the *Diagnostic and Statistical Manual* (DSM-I) [7], it listed all the conditions psychiatrists then considered to be a mental disorder. DSM-I classified “homosexuality” as a “sociopathic personality disturbance.” In DSM-II, published in 1968 [8], homosexuality was reclassified as a “sexual deviation.” 

## 6. The Sexologists 

As psychiatrists, physicians, and psychologists tried to “cure” homosexuality, sex researchers of the mid-20th century instead studied a wider spectrum of individuals that included non-patient populations. Psychiatrists and other clinicians drew conclusions from a skewed sample of patients seeking treatment for homosexuality or other difficulties and then wrote up their findings of this self-selected group as case reports. Some theories about homosexuality were based on studies of prison populations. Sexologists, on the other hand, did field studies in which they went out and recruited large numbers of non-patient subjects in the general population.

The most important research in this area was that of Alfred Kinsey and his collaborators, published in two headline-generating reports [22,23]. The Kinsey reports, surveying thousands of people who were not psychiatric patients, found homosexuality to be more common in the general population than was generally believed, although his now-famous “10%” statistic is today believed to be closer to 1%–4% [46]. This finding was sharply at odds with psychiatric claims of the time that homosexuality was extremely rare in the general population. Ford and Beach’s [47] study of diverse cultures and of animal behaviors, confirmed Kinsey’s view that homosexuality was more common than psychiatry maintained and that it was found regularly in nature. In the late 1950s, Evelyn Hooker [24], a psychologist, published a study in which she compared psychological test results of 30 gay men with 30 heterosexual controls, none of whom were psychiatric patients. Her study found no more signs of psychological disturbances in the gay male group, a finding that refuted psychiatric beliefs of her time that all gay men had severe psychological disturbances.

## 7. The 1973 APA Decision

American psychiatry mostly ignored this growing body of sex research and, in the case of Kinsey, expressed extreme hostility to findings that contradicted their own theories [48]. It should be further noted that some mid-20th century homophile (gay) activist groups accepted psychiatry’s illness model as an alternative to societal condemnation of homosexuality’s “immorality” and were willing to work with professionals who sought to “treat” and “cure” homosexuality. Other gay activists, however, forcefully rejected the pathological model as a major contributor to the stigma associated with homosexuality. It was this latter group that brought modern sex research theories to the attention of APA. In the wake of the 1969 Stonewall riots in New York City [49], gay and lesbian activists, believing psychiatric theories to be a major contributor to anti-homosexual social stigma, disrupted the 1970 and 1971 annual meetings of the APA.

As Bayer [1] has noted, factors both outside and within APA would lead to a reconceptualization of homosexuality’s place in the DSM. In addition to research findings from outside psychiatry, there was a growing anti-psychiatry movement [50], not to mention cultural studies critics who held medicine’s history of diagnostic excess up for ridicule, citing the example of *drapetomania,* a 19th century “disorder of slaves who have a tendency to run away from their owner due to an inborn propensity for wanderlust” [51], (p. 357). 

There was also an emerging generational changing of the guard within APA comprised of younger leaders urging the organization to greater social consciousness [2]. A very few psychoanalysts like Judd Marmor [5,52] were also taking issue with psychoanalytic orthodoxy regarding homosexuality. However, the most significant catalyst for diagnostic change was gay activism. 

Gay activist protests succeeded in getting APA’s attention and led to unprecedented educational panels at the group’s next two annual meetings. A 1971 panel, entitled “Gay is Good,” featured gay activists Frank Kameny and Barbara Gittings explaining to psychiatrists, many who were hearing this for the first time, the stigma caused by the “homosexuality” diagnosis [53,54,55]. Kameny and Gittings returned to speak at the 1972 meeting, this time joined by John Fryer, M.D. Fryer appeared as Dr. H Anonymous, a “homosexual psychiatrist” who, given the realistic fear of adverse professional consequences for coming out at that time, disguised his true identity from the audience and spoke of the discrimination gay psychiatrists faced in their own profession [1,2].

While protests and panels took place, APA engaged in an internal deliberative process of considering the question of whether homosexuality should remain a psychiatric diagnosis. This included a symposium at the 1973 APA annual meeting in which participants favoring and opposing removal debated the question, “Should Homosexuality be in the APA Nomenclature?” [56]. The Nomenclature Committee, APA’s scientific body addressing this issue also wrestled with the question of what constitutes a mental disorder. Robert Spitzer, who chaired a subcommittee looking into the issue, “reviewed the characteristics of the various mental disorders and concluded that, with the exception of homosexuality and perhaps some of the other ‘sexual deviations’, they all regularly caused subjective distress or were associated with generalized impairment in social effectiveness of functioning” [57], (p. 211). Having arrived at this novel definition of mental disorder, the Nomenclature Committee agreed that homosexuality *per se* was not one. Several other APA committees and deliberative bodies then reviewed and accepted their work and recommendations. As a result, in December 1973, APA’s Board of Trustees (BOT) voted to remove homosexuality from the DSM.

Psychiatrists from the psychoanalytic community, however, objected to the decision. They petitioned APA to hold a referendum asking the entire membership to vote either in support of or against the BOT decision. The decision to remove was upheld by a 58% majority of 10,000 voting members. 

It should be noted that psychiatrists did *not* vote, as is often reported in the popular press, on whether homosexuality should remain a diagnosis. What APA members voted on was to either “favor” or “oppose” the APA Board of Trustees decision and, by extension, the scientific process they had set up to make the determination [1], (p. 148). Further, opponents of the 1973 removal have repeatedly tried to discredit the referendum’s outcome by declaring, “science cannot be decided by a vote” [58]. However they usually neglect to mention that those favoring retention of the diagnosis were the ones who petitioned for a vote in the first place. In any event, in 2006 the International Astronomical Union voted on whether Pluto was a planet [59,60], demonstrating that even in a hard science like astronomy, interpretation of facts are always filtered through human subjectivity.

In any event, the events of 1973 did not immediately end psychiatry’s pathologizing of some presentations of homosexuality. For in “homosexuality’s” place, the DSM-II contained a new diagnosis: Sexual Orientation Disturbance (SOD). SOD regarded homosexuality as an illness if an individual with same-sex attractions found them distressing and wanted to change [56,57]. The new diagnosis legitimized the practice of sexual conversion therapies (and presumably justified insurance reimbursement for those interventions as well), even if homosexuality *per se* was no longer considered an illness. The new diagnosis also allowed for the unlikely possibility that a person unhappy about a heterosexual orientation could seek treatment to become gay [61].

SOD was later replaced in DSM-III [9] by a new category called “Ego Dystonic Homosexuality” (EDH) [57]. However, it was obvious to psychiatrists more than a decade later that the inclusion first of SOD, and later EDH, was the result of earlier political compromises and that neither diagnosis met the definition of a disorder in the new nosology. Otherwise, all kinds of identity disturbances could be considered psychiatric disorders. “Should people of color unhappy about their race be considered mentally ill?” critics asked. What about short people unhappy about their height? Why not ego-dystonic masturbation [62]? As a result, ego-dystonic homosexuality was removed from the next revision, DSM-III-R, in 1987 [10]. In so doing, the APA implicitly accepted a normal variant view of homosexuality in a way that had not been possible fourteen years earlier [63].

## 8. Conclusions

APA’s 1973 diagnostic revision was the beginning of the end of organized medicine’s official participation in the social stigmatization of homosexuality. Similar shifts gradually took place in the international mental health community as well. In 1990, the World Health Organization removed homosexuality per se from the International Classification of Diseases (ICD-10) [64]. As a consequence, debates about homosexuality gradually shifted away from medicine and psychiatry and into the moral and political realms as religious, governmental, military, media, and educational institutions were deprived of medical or scientific rationalization for discrimination. 

As a result, cultural attitudes about homosexuality changed in the US and other countries as those who accepted scientific authority on such matters gradually came to accept the normalizing view. For if homosexuality was no longer considered an illness, and if one did not literally accept biblical prohibitions against it, and if gay people are able and prepared to function as productive citizens, then what is wrong with being gay? Additionally, if there is nothing wrong with being gay, what moral and legal principles should the larger society endorse in helping gay people openly live their lives?

The result, in many countries, eventually led, among other things, to (1) the repeal of sodomy laws that criminalized homosexuality; (2) the enactment of laws protecting the human rights of lesbian, gay, bisexual and transgender (LGBT) people in society and the workplace; (3) the ability of LGBT personnel to serve openly in the military; (4) marriage equality and civil unions in an ever growing number of countries; (5) the facilitation of gay parents’ adoption rights; (6) the easing of gay spouses’ rights of inheritance; and (7) an ever increasing number of religious denominations that would allow openly gay people to serve as clergy. 

Most importantly, in medicine, psychiatry, and other mental health professions, removing the diagnosis from the DSM led to an important shift from asking questions about “what causes homosexuality?” and “how can we treat it?” to focusing instead on the health and mental health needs of LGBT patient populations [65]. 
